# Causal impact of DNA methylation on refracture in elderly individuals with osteoporosis – a prospective cohort study

**DOI:** 10.1186/s12891-024-07521-y

**Published:** 2024-06-03

**Authors:** Bingtao Wen, Yaning Zhang, Jianhua He, Lei Tan, Guanggui Xiao, Zunliang Wang, Wei Cui, Bingxuan Wu, Xianhai Wang, Lei He, Ming Li, Zhongjiao Zhu, Dacheng Sang, Changqing Zeng, Peilin Jia, Fan Liu, Tianzi Liu

**Affiliations:** 1 Department of Orthopedics, Peking University International Hospital, Beijing, China; 2 CAS Key Laboratory of Genomic and Precision Medicine, Beijing Institute of Genomics, Chinese Academy of Sciences and China National Center for Bioinformation, Beijing, China; 3 Institute of Biomedical Research, Henan Academy of Sciences, Zhengzhou, Henan China; 4 Department of Rehabilitation, the Second Medical Center of Chinese People’s Liberation Army General Hospital, Beijing, China; 5 Beijing University of Posts and Telecommunications, Beijing, China; 6 Department of Orthopedics, Beijing Tiantan Hospital, Capital Medical University, Beijing, China; 7 Department of Orthopedics, Changping District Hospital, Beijing, China; 8 Department of Orthopedics, Tengzhou Central People’s Hospital, Tengzhou, Shandong China; 9 Department of Orthopaedic Surgery, Yijishan Hospital, The First Affiliated Hospital of Wannan Medical College, Wannan Medical College, Wuhu, Anhui China; 10 Department of Forensic Sciences, College of Criminal Justice, Naif Arab University for Security Sciences, Riyadh, Kingdom of Saudi Arabia; 11 CAS Key Laboratory of Computational Biology, Shanghai Institute of Nutrition and Health, Chinese Academy of Sciences, Shanghai, China

**Keywords:** Osteoporosis, Vertebral compression fracture, Vertebral augmentation, Refracture, DNA methylation

## Abstract

**Background:**

Osteoporotic vertebral compression fractures (OVCF) in the elderly increase refracture risk post-surgery, leading to higher mortality rates. Genome-wide association studies (GWAS) have identified susceptibility genes for osteoporosis, but the phenotypic variance explained by these genes has been limited, indicating the need to explore additional causal factors. Epigenetic modifications, such as DNA methylation, may influence osteoporosis and refracture risk. However, prospective cohorts for assessing epigenetic alterations in Chinese elderly patients are lacking. Here, we propose to conduct a prospective cohort study to investigate the causal network of DNA polymorphisms, DNA methylation, and environmental factors on the development of osteoporosis and the risk of refracture.

**Methods:**

We will collect vertebral and peripheral blood from 500 elderly OVCF patients undergoing surgery, extract DNA, and generate whole genome genotype data and DNA methylation data. Observation indicators will be collected and combined with one-year follow-up data. A healthy control group will be selected from a natural population cohort. Epigenome-wide association studies (EWAS) of osteoporosis and bone mineral density will be conducted. Differential methylation analysis will compare candidate gene methylation patterns in patients with and without refracture. Multi-omics prediction models using genetic variants and DNA methylation sites will be built to predict OVCF risk.

**Discussion:**

This study will be the first large-scale population-based study of osteoporosis and bone mineral density phenotypes based on genome-wide data, multi-time point methylation data, and phenotype data. By analyzing methylation changes related to osteoporosis and bone mineral density in OVCF patients, the study will explore the feasibility of DNA methylation in evaluating postoperative osteoporosis intervention effects. The findings may identify new molecular markers for effective anti-osteoporosis treatment and inform individualized prevention and treatment strategies.

**Trial registration:**

chictr.org.cn ChiCTR2200065316, 02/11/2022.

**Supplementary Information:**

The online version contains supplementary material available at 10.1186/s12891-024-07521-y.

## Background

Osteoporotic vertebral compression fractures (OVCF) are the most common fracture type in patients with osteoporosis, accounting for about half of osteoporosis-related fractures [1]. These fractures are associated with high morbidity and mortality [2], and vertebral augmentation is the standard operation for their treatment. While vertebral augmentation has significant effects in relieving pain and restoring vertebral height [3], it cannot fundamentally cure the fracture and prevent recurrence. Furthermore, the literature reports that the rate of recurrence of OVCF after vertebroplasty is high [4], ranging from 6.2% to 51.8% [5]. The debate on the risk factors of refracture after vertebral augmentation has lasted for nearly 20 years, but it is still inconclusive.

Osteoporosis is a complex disease caused by both genetic and environmental factors. While hundreds of genes and loci associated with osteoporotic fractures or bone mineral density have been identified through genome-wide association studies (GWAS), these can only explain a small proportion of the variation in the disease and its manifestations. For example, the 20 genes found in *GEFOS-1* explain only 5% of the phenotypic variation [6]. The hundreds of loci identified by the GWAS of bone mineral density based on UK biobank samples could only explain 12% to 20% of the phenotypic variation [7]. Therefore, the susceptibility genetic loci of osteoporosis found by GWAS alone cannot achieve accurate prediction of phenotypic changes, and even the prediction model based on a large number of genetic loci cannot achieve the accuracy required for practical applications in clinics.

Epigenetic modifications, including DNA methylation and histone modification, affect cell differentiation and development by affecting the structure and expression of genes, thereby regulating the genetic risk and environmental influence of various complex phenotypes and diseases [8]. Therefore, DNA methylation modification is considered as a bridge connecting genotypes and individual phenotypes. Epigenome-wide association studies (EWAS) of bone mineral density have discovered methylation sites playing a significant role in the pathogenesis of osteoporosis [9–15]. Furthermore, previous studies have revealed that exercise intervention for osteoporosis may achieve its effects by modulating the expression of bone metabolism genes via alterations in DNA methylation levels [16–18].

The objective of this study is to investigate the genetic and epigenetic markers associated with osteoporosis and bone mineral density, and their impact on refracture risk among elderly patients. We aim to collect vertebral body blood and peripheral blood samples from 500 patients with OVCF who will undergo detailed clinical assessments, genome-wide genotype and methylation profiling at baseline. Following the surgical intervention, these patients will be followed up for 1 year to monitor refracture events, during which clinical and genome-wide methylation data will be collected again. These valuable data will enable us to identify genetic and epigenetic markers that can predict OVCF and refracture risk in the elderly and develop accurate prediction models.

## Methods

### Recruitment of patients

**Fig. 1:**
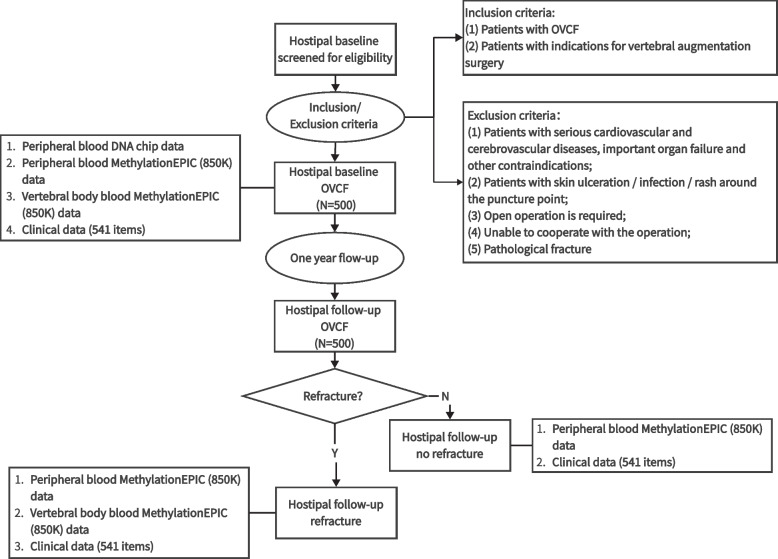
Flowchart of this trial. OVCF: osteoporotic vertebral compression fracture

**Fig. 2:**
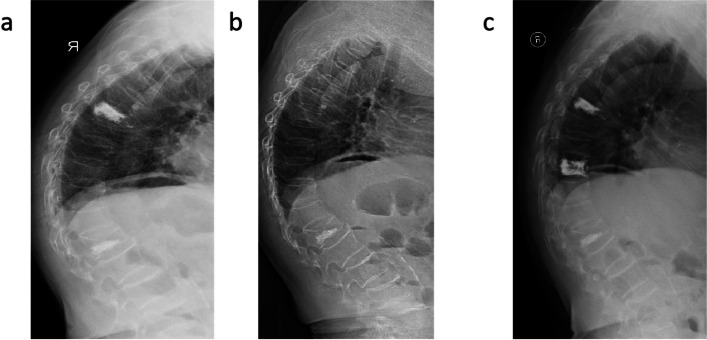
Refracture in elderly individuals with osteoporosis who undergo vertebral augmentation operation. A patient, xx, female, 85 years old, underwent three vertebral augmentation operations within 2 years due to osteoporotic vertebral compression fracture. **a** The first vertebral augmentation operation (L1 vertebral body) was performed in June 2018. **b** The second vertebral augmentation operation (Thoracic 7 vertebra body) was performed in October 2019. **c** The third vertebral augmentation operation (Thoracic 10 vertebra body) was performed in July 2020

This will be a prospective multicenter cohort study conducted in 5 medical centers, including Peking University International Hospital, Beijing Tiantan Hospital affiliated to Capital Medical University, Beijing Changping District Hospital, Tengzhou Central People’s Hospital, and Linfen People’s Hospital. The study was approved by the Biomedical Ethics Committee of Peking University International Hospital (batch number: 2022-KY-0010-01). Recruitment of elderly patients with OVCF after vertebral augmentation and those without refracture will take place from December 2022 to December 2023. All participants will provide informed consent. The flow chart is presented in Fig. 1. An example of refracture in elderly individuals with osteoporosis who undergo vertebral augmentation is shown in Fig. 2.

### Sample size

The power of EWAS is essential for detecting differentially methylated positions (DMPs) between cases and controls. Similar to GWAS, the power of EWAS depends on four key factors, including study design, sample size, effect size, and burdon of multiple test correction. Previous simulation analysis has shown that a sample of 500 cases and 500 controls would achieve 80% power to detect differential methylation at epigenome-wide significance (*P* < 1E-6) with a methylation odds ratio of 1.28 or mean methylation differences of 5% in case–control designs of EWAS (cite the original pubication) [19]. Therefore, we aim to recruit 500 elderly patients with OVCF who underwent vertebral augmentation surgery, as well as 500 healthy controls matched for age, gender, and race from the CAS cohort who did not develop OVCF or osteoporosis. This sample size will provide adequate statistical power for detecting significant differences in DNA methylation levels between cases and controls and enable us to identify epigenetic markers associated with refracture after vertebral augmentation surgery for OVCF.

### Eligibility criteria

(1) Elderly patients aged ≥ 60 years who underwent vertebral augmentation for OVCF and experienced recurrent fractures after the initial surgery; (2) Elderly patients aged ≥ 60 years with bone mineral density T ≤ -2.5.

### Exclusion criteria

(1) Patients with severe cardiovascular and cerebrovascular diseases, including but not limited to myocardial infarction, stroke, and severe arrhythmias; (2) Patients with significant organ failure, such as end-stage renal disease requiring dialysis, or liver failure requiring transplantation; (3) Patients with contraindications to vertebral augmentation surgery, such as coagulopathy or anticoagulant therapy that cannot be safely managed; (4) Patients with skin ulceration, infection, or rash around the puncture point that would increase the risk of complications during surgery; (5) Patients requiring open surgery for their OVCF, as this may indicate a more complex or severe fracture that could affect the study outcomes; (6) Patients who are unable to cooperate with surgery due to cognitive impairment, psychiatric illness, or other reasons that would make the procedure unsafe or unsuccessful; (7) Patients with a history of pathological fractures, indicating underlying bone disease or malignancy.

### Inclusion criteria for the control group

(1) Diagnosed elderly patients (aged 60 years and older) with OVCF who underwent vertebral augmentation and did not experience refracture within a specified follow-up period after the initial surgery; (2) Elderly patients aged 60 years and older with a bone mineral density T-score of -2.5 or lower, indicating osteoporosis.

### Exclusion criteria for the control group

(1) Patients who underwent vertebral augmentation due to a pathological fracture caused by underlying bone disease or malignancy; (2) Elderly patients with OVCF receiving conservative treatment only, without undergoing vertebral augmentation surgery.

### Survey contents and methods (observation indicators)

The study includes eight categories of observation indicators and a total of 541 data items, as follows:

1. Basic information: 21 data items, including age, gender, BMI, etc.
2. Habit characteristics: 34 items in total, including eating habits, exercise habits, family activities, medication, and behavioral risk factors.
3. Past medical history: 13 data items.
4. Comorbidities/medication: 29 items.
5. Diagnostic tests: 253 data items, including routine blood tests, coagulation tests, biochemistry, bone metabolism, sex hormones, white blood cell count, skin, pancreas and serum tests, thyroid function tests, tumor markers, imaging, functional score, color Doppler ultrasound, echocardiography, electrocardiogram, sarcopenia, infection index, and SF-36 quality of life (Table S1 and S2).
6. Treatment plan: 36 data items.
7. Postoperative follow-up: 139 data items, including follow-up table, functional score, bone metabolism, and SF-36 quality of life.
8. Adverse events: 16 data items.

The follow-up plan includes two parts:

1. Patients will enter the data independently using their mobile phones, with real-time network supervision to ensure accurate data entry.
2. Doctors will conduct monthly or regular telephone follow-ups to guide postoperative rehabilitation, which will last for 1 year.

The clinical data of all OVCF patients will be collected and input into a structured database through an established clinical research platform (Table 1). The platform will classify and manage the data into eight categories: basic information, habit characteristics, past medical history, comorbidities and medication, diagnostic examination, treatment plan, postoperative follow-up (Table S3), and adverse events. The data will be collected from the hospital, including medical history, physical examination, hematology, and imaging data, were directly uploaded through photos, and the research platform staff sorted the data into the corresponding categories. The data collected outside the hospital will be uploaded through a two-dimensional code automatic real-time clocking method, and the doctors supervised the data entry, which will be then sorted by the research platform staff. To analyze bone formation and resorption markers, blood samples will be analyzed using laboratory techniques such as ELISA to measure the levels of specific markers associated with bone metabolism. The results will be interpreted by comparing the marker levels to established reference ranges or normal values, aiding in the assessment of bone health and related conditions.

**Table 1:** Schedule of enrolment and assessments

	**Treatment stage**	**Pre-operation examination**	**One year after the operation**
Patient information	+		
Eating habits	+		
Exercise habit	+		
Family activities	+		
Medication	+		
Behavioral risk factors	+		
Routine blood test		+	
Seven items of blood coagulation test		+	
Biochemistry test		+	
Four items of bone metabolism test		+	+
Six items of sex hormone test		+	
Interleukin-Growth Hormone-Cortisol-Insulin-Serum		+	
Seven items of thyroid function		+	
Imaging examination		+	
Function score		+	+
Colour ultrasonic diagnosis		+	
Echocardiogram		+	
Sarcopenia		+	
Electrocardiogram		+	
Tumor marker		+	
Infection indicators		+	
SF-36 life quality		+	+
Follow-up table			+

For the whole-genome microarray genotyping data, we will use the Infinium Asian Screening Array + MultiDisease -24 (ASA-MD) genotyping chip. Genome Studio software will be used for quality control of raw data. Samples with a call rate less than 99% and SNPs with a call rate less than 98% will be filtered out. After quality control, PLINK software will be used for further processing. Samples with kinship will be excluded by calculating the Identity-By-Descent (IBD) and inbreeding coefficient (F). Imputation and preprocessing will be performed based on the 1000 genomes of the East Asian population and the Han population genome of the previous study. SNPs that do not meet the Hardy–Weinberg equilibrium (HWE ≥ 0.001) and minor allele frequency (MAF ≥ 0.01) will be excluded.

For the genome-wide methylation beadchip data, DNA from vertebral and peripheral blood samples stored at -80 °C will be bisulfite converted, and the Infinium MethylationEPIC BeadChips (Illumina) will be used for genome-wide DNA methylation profiling. BeadChips will be processed according to the manufacturer’s protocol, and then imaged using the iScan Microarray Scanner (Illumina). The resulting idat files will be processed using the CHAMP Bioconductor package. Samples with missing probes greater than 10% and probes missing in samples more than 20% will be removed, and KNN imputation will be carried out for the rest. Probes with less than three beads in more than 5% of samples and non-CpG, cross-reactive, or non-specific probes will also be removed. Probes mapping on the sex chromosomes and contained SNPs will also be removed. After checking sample outliers by MDS plots and beta values distribution of Type-I and Type-II, data normalization will be performed using BMIQ. Samples with genotype data will be kept, and methylation beta values will be transformed into M values.

### Biomarker identification and prediction analysis

The GWAS of bone mineral density will be carried out in PLINK using linear regression model, assuming an additive allele effect. Covariates for age, gender, drug usage and the first five principal components to account for residual population structure will be also included in the model. The genomic control inflation factor will be used to adjust the *p*-values. The adjusted *p*-values equal to or smaller than 5E − 8 will be considered as genoome-wide significant. We will conduct a GWAS using case–control design to invesgate the genetic variants associated with osteoporosis.

This study will use whole-genome methylation data from vertebral and peripheral blood samples of 500 osteoporosis patients with recurrent fractures after vertebral augmentation and 1000 natural population controls from the Chinese Academy of Sciences. To identify differentially methylated positions (DMPs) between patients with and with out refracture, we will perform an epigenome-wide association study (EWAS) and meQTL analysis. Patients with refracture who underwent vertebral augmentation will be selected as cases, and an equal number of patients without refracture will be selected as controls based on age range and epidemiological indicators such as BMI, disease, and medication history. We will analyze changes in methylation levels of osteoporosis- and bone mineral density-related sites in elderly patients with refracture within one year after surgery. The R language DMRfinder package will be used for analysis, and functional annotation of the identified DMPs and differentially methylated regions (DMRs) will be performed.

To predict BMD and OVCF risk, we will use multiple linear regression and multiple logistic regression models, respectively. We will include sex and age as predictors along with independent BMD-related genetic variants identified in previous studies and methylation sites identified in this study. Fracture risk score will also be evaluated based on age, gender, bone mineral density (BMD), previous fracture history, family history, and other risk factors. Prediction accuracy will be estimated using the R-squared correlation (R2) for BMD and the area under the curve (AUC) for OVCF risk. Sensitivities and specificities will be calculated using confusion matrices, where the predicted probability greater than a certain threshold (t) will be considered the predicted shape type, and t will be optimized to maximize the sum of sensitivity and specificity.

### Clinical implications/impact of the study

The systematic analysis of molecular mechanisms underlying osteoporosis and bone mineral density phenotypes is of great importance, and this study aims to explore cross-omics molecular markers that affect these phenotypes based on multi-omics data and the degree of physiological aging. The research objectives of this study include:

1. Conducting genome-wide association analysis and genome-wide epigenetic association analysis of osteoporosis and bone mineral density to identify genetic and epigenetic molecular markers related to these phenotypes and to deeply analyze their molecular mechanisms.
2. Evaluating the feasibility of DNA methylation in assessing the effect of postoperative intervention for osteoporosis by analyzing changes in methylation levels of bone mineral density related sites in elderly patients with refracture after vertebral augmentation for OVCF within one year after surgery.
3. Improving the risk prediction model of osteoporosis by considering multiple factors such as genetic variation and environmental factors, which can provide a scientific basis for the molecular classification and prevention of related diseases and serve as a paradigm for biomedical researchers conducting multi-omics life data research.

The results of this study can provide valuable insights into the molecular mechanisms of osteoporosis and bone mineral density phenotypes, which can facilitate the development of effective interventions and personalized treatments. The findings can also contribute to the establishment of a risk prediction model for osteoporosis that takes into account genetic and environmental factors, which can help identify individuals at high risk for developing osteoporosis and enable targeted preventive measures. Overall, this study has the potential to make significant contributions to the field of osteoporosis research and improve clinical practice in the management of related diseases.

### Advanced features of this study

1. To ensure data quality, we have established a special OVCF real-world research database system that supports data operation traceability, automatic logical verification, and other functions based on the ALCOA principle.
2. We have assigned a special person to inspect the organization and implementation of the study, verify the data, and ensure accurate, timely, and complete data collection through quality control and assurance work meetings attended by all implementation units.
3. To control the risk of loss to follow-up, we have implemented a patient data entry method and set up a special person to supervise the data entry. Follow-up personnel (doctors/nurses) will also follow up on patients’ data entry by monthly or regular telephone follow-up.
4. To control patient compliance bias, we have established a unified clinical research platform for observation indicators such as out-of-hospital medication and dietary habits, and jointly set up personalized anti-osteoporosis programs with the department of endocrinology, rehabilitation, and nutrition to increase patient compliance. The study protocol was designed as a real-world study, and the final analysis was divided into groups according to the completion of the protocol to make the results closer to the clinical situation and increase the credibility of the study.
5. We have implemented strict biobank management, ensuring the samples were only used for this study, destroyed after research, and information leakage was strictly controlled.
6. Our study utilizes a multi-omics approach, incorporating whole genome sequencing and genome-wide methylation analysis to explore the interaction between genetics, epigenetics, and the environment in osteoporosis and bone mineral density phenotypes. This provides a more comprehensive understanding of the molecular mechanisms underlying these conditions.
7. We have included a control group of 1000 healthy individuals from the Chinese Academy of Sciences natural population cohort, providing a larger and more diverse sample for comparison and analysis.
8. We are exploring the feasibility of DNA methylation as a tool for evaluating the effect of postoperative intervention for osteoporosis by analyzing changes in the methylation levels of bone mineral density-related sites in elderly patients with refracture after vertebral augmentation for OVCF within one year after surgery.
9. We are improving the risk prediction model for osteoporosis by incorporating multiple factors such as genetic variation and environmental factors, providing a more personalized approach to prevention and treatment.
10. Our study is conducted in multiple medical centers, increasing the diversity of patient populations and enhancing the generalizability of our findings.

## Discussion

OVCF is a debilitating disease that affects the elderly population, and effective anti-osteoporosis treatment is crucial in preventing refracture and improving quality of life. In this study, we aimed to investigate the genetic and epigenetic mechanisms underlying osteoporosis and bone mineral density and explore the feasibility of DNA methylation in evaluating postoperative intervention for OVCF patients. Our study overcame previous limitations in sample size, cohort characteristics, and lack of healthy controls and utilized a big data research approach to understand the complex interactions between genetics, epigenetics, and the environment.

By conducting genome-wide association analysis and epigenetic association analysis of osteoporosis and bone mineral density, we identified genetic and epigenetic markers related to these phenotypes and deepened our understanding of the underlying molecular mechanisms. Our findings can provide a scientific basis for personalized prevention, treatment, and medication guidance in osteoporosis.

Moreover, we explored the potential of DNA methylation as a biomarker for evaluating the effect of postoperative intervention for osteoporosis by analyzing the changes in methylation levels of bone mineral density-related sites in elderly patients with refracture after vertebral augmentation. This approach may provide valuable insight into the effectiveness of treatment and help guide clinical decision-making.

To ensure the accuracy, completeness, and consistency of our data, we implemented quality control measures throughout the study process, including a specialized OVCF real-world research database system, data verification, risk control of loss to follow-up, patient compliance bias control, and biobank management.

In conclusion, our study provides new insights into the molecular mechanisms of osteoporosis and bone mineral density and demonstrates the potential of DNA methylation as a biomarker for evaluating the effectiveness of postoperative intervention for OVCF patients. Our findings can inform personalized prevention, treatment, and medication guidance and contribute to the development of multi-omics life data research.

### Supplementary Information

Supplementary Material 1.

## Data Availability

The final dataset will be kept by the principal investigator and can be supplied on reasonable request. The information will be kept for 10 years and will not be disclosed to a third party. After the study is completed, the data will be made available on request from the corresponding author.

## Abbreviations

ALCOA: Attributable, Legible, Contemporaneous, Original, Accurate
ASA-MD: Asian Screening Array + MultiDisease
AUC: Area Under the Curve
BMD: Bone Mineral Density
BMI: Body Mass Index
BMIQ: Beta MIxture Quantile dilation
CHAMP: Chip Analysis Methylation Pipeline
DNA: Deoxyribonucleic Acid
DMR: Differentially Methylated Region
ELISA: Enzyme-Linked Immunosorbent Assay
EWAS: Epigenome-Wide Association Study
GEFOS: Genetic Factors for Osteoporosis Consortium
GWAS: Genome-Wide Association Study
HWE: Hardy-Weinberg Equilibrium
IBD: Identity-By-Descent
KNN: K-Nearest Neighbors
MAF: Minor Allele Frequency
MDS: Multidimensional Scaling
meQTL: Methylation Quantitative Trait Loci
OVCF: Osteoporotic Vertebral Compression Fracture
PLINK: Whole Genome Association Analysis Toolset
R2: R-squared
SF-36: 36-Item Short Form Survey
